# Double Heterozygosity for Germline Mutations in Chinese Breast Cancer Patients

**DOI:** 10.3390/cancers16142547

**Published:** 2024-07-15

**Authors:** Ava Kwong, Cecilia Y. S. Ho, Chun-Hang Au, Edmond S. K. Ma

**Affiliations:** 1 Department of Surgery, The University of Hong Kong, Hong Kong SAR, China; 2 Department of Surgery, Hong Kong Sanatorium & Hospital, Hong Kong SAR, China; 3 Hong Kong Hereditary Breast Cancer Family Registry, Hong Kong SAR, China; 4 Department of Molecular Pathology, Hong Kong Sanatorium & Hospital, Hong Kong SAR, China

**Keywords:** *BRCA*, hereditary breast cancers, Chinese, double heterozygosity

## Abstract

**Simple Summary:**

About 5–10% of breast cancers are related to heredity. Very often, affected individuals will carry only a single mutation defect in any of the hereditary breast and ovarian cancer syndrome (HBCO)-related genes. Breast cancer patients who are double heterozygous (DH) for different HBCO-related genes are rare. In this study, we provide real-world data for the Chinese population based on our high-risk referral patients from the Hong Kong Hereditary Breast Cancer Family Registry. DH germline mutations were identified in nine patients (0.25%) and associated with a higher prevalence of bilateral breast cancers in the Chinese population compared to other populations. A more stringent surveillance program and possibly a more aggressive treatment plan for all DH families could be tailored to specific family needs in the local area.

**Abstract:**

Double pathogenic mutations occurring in an individual are considered a rare event. The introduction of a multiple-gene panel at Hong Kong Hereditary Breast Cancer Family Registry has allowed the identification of pathogenic variants in multiple genes, providing more information on clinical management and surveillance to the proband and their family members. Breast cancer patients who are double heterozygous (DH) for different hereditary breast and ovarian cancer syndrome (HBCO)-related genes were identified from a cohort of 3649 Chinese patients. Nine patients (0.25%) were observed to have germline DH mutations in *ATM*, *BRCA1*, *BRCA2*, *BRIP1*, *CDH1*, *CHEK2*, *MSH6*, *PALB2*, and *TP53*. Three probands were diagnosed with unilateral breast cancer, two patients were diagnosed with bilateral breast cancer, and four patients had multiple primary cancers. The median age for breast cancer diagnosis was an early age of 36 years. Chinese DH carriers did not show worse phenotypes or have a significantly downhill clinical presentation. However, seven out of nine (77.8%) of our DH carriers harbored a *BRCA1* mutation, and four of them (44.4%) developed bilateral breast cancer, suggesting Chinese DH individuals may have a higher chance of having bilateral breast cancer than other populations (*p* = 0.0237).

## 1. Introduction

Hereditary breast and ovarian cancer syndrome is an inherited cancer-predisposition syndrome, predominantly caused by mutations in the *BRCA* genes. Women carrying *BRCA1* and *BRCA2* pathogenic variants have a significant lifetime risk of breast and ovarian cancers, with rates of up to 85% and 65%, respectively [1]. Men with these mutations have a 1% greater lifetime risk of breast cancer than male non-carriers [2], in addition to a risk of developing prostate carcinomas.

The vast majority of *BRCA1* and *BRCA2* mutation carriers are simple heterozygotes (SH) for *BRCA1* or *BRCA2*. Double heterozygosity (DH) for tumor-predisposing germline mutations is rare but has been observed in recognized founder populations, such as Ashkenazi Jews [3,4,5,6,7,8]. From worldwide studies, DH occurs in 0.09–0.36% of index cases, of which 0.22–0.87% are proven *BRCA* mutation carriers, rising to 1.8% in Ashkenazi Jews. At least one of the detected mutations is usually a founder mutation from Ashkenazi Jews, mainly c.68_69delAG; p.(Glu23Valfs) and c.5266dup; p.(Gln1756fs) in the *BRCA1* gene and c.5946del; p.(Ser1982fs) in the *BRCA2* gene [9]. In a few recurrent *BRCA1* alleles, the founder effect has also been observed in several Slavic countries [10,11,12]. Furthermore, Slavic breast cancer patients have a high mutation frequency of *BRCA1*: c.4035del; p.(Glu1346fs); *CHEK2:* c.1100del; p.(Thr367fs) and deletion of exon 9–10; *BLM*: c.1642C>T; p.(Gln548Ter); *ATM*: c.5932G>T; p.(Glu1978Ter); and *NBN:* c.657_661del; p.(Lys219fs) mutations [10,11,12,13,14], resulting in a higher frequency of DH identified in these populations.

DH mutations are uncommon, particularly in other non-Ashkenazi and Slavic individuals. A recent study on Brazilians identified 1.2% DH cases from among a cohort of 1156 early-onset breast cancer patients and no significant differences in age of BC onset and risk for bilateral BC in DH carriers of simple heterozygote (SH) mutations [15]. However, in a Caucasian population, the proportion of DH BRCA females identified was 0.1%, and they might develop breast cancer at an earlier age and have more severe disease than SH BRCA mutation carriers [16]. Only a few DH cases have been reported in the Asian population [17,18,19], and the corresponding cancer risk is still unknown. DH of *BRCA* mutations was identified in 93 females from 32,295 *BRCA1/2* mutation carriers listed in the CIMBA database [20].

To characterize the nature of DH and its clinical phenotypes in the Chinese populations, we systematically evaluated the frequency and clinical characteristics of DH and compared them with those of carriers of SH mutation. This was conducted in order to create a more suitable surveillance program and treatment plan for DH families.

## 2. Methods

### 2.1. Participants and Selection Criteria

Germline mutation screening was performed on 3649 high-risk Chinese breast cancer patients recruited through the Hong Kong Hereditary Breast Cancer Family Registry from March 2007 to August 2022. Individuals were included in this study if they met any of the following criteria: (1) they had been diagnosed with breast cancer at any age and had at least one first- or second-degree relative with breast and/or ovarian cancer, regardless of age; (2) they had been diagnosed with breast cancer at or before 45 years of age; (3) they had bilateral breast cancer; (4) they had triple-negative breast cancer; or (5) they were male with breast cancer. All participants recruited gave their consent for the study, and the research was conducted in accordance with the Declaration of Helsinki.

### 2.2. DNA Extraction and Sequencing

Genomic DNA was pooled and sequenced with a 30-gene panel (Color Genomics Laboratory, Burlingame, CA, USA) or a 93-gene DHS-001Z human breast cancer panel (Qiagen, Hilden, Germany) using MiSeq or NextSeq (Illumina, San Diego, CA, USA) instruments after extraction from peripheral blood using the QIAamp DNA Blood Mini Kit or the QIAsymphony DNA Mini Kit (Qiagen, Hilden, Germany) according to the manufacturer’s instructions. The minimum sequencing depth and median coverage were typically 50-fold with 200–300X. All detected pathogenic variants were further confirmed and validated via conventional Sanger bi-directional DNA sequencing.

### 2.3. Variant Interpretation and Annotation

Variant-calling bioinformatics was performed, and paired sequencing reads were mapped to human reference genome sequence GRCh37/hg19, as previously described [21,22]. Variants with a minor allele frequency of at least 1%, as reported by the 1000 Genomes Project [23], were excluded from manual variant curation. The variants were described according to the recommendations of the Human Genome Variation Society (HGVS) nomenclature (http://www.HGVS.org/varnomen, (accessed on 4 July 2022)) and further cross-checked with the Mutalyzer Name Checker (http://mutalyzer.nl).

### 2.4. Statistical Analysis

Fisher’s exact test was used to analyze the relationship between clinicopathological variables and mutation status. The threshold of statistical significance was set at a *p*-value of <0.05 for all analyses. Data analyses were conducted using the statistical software program R (version 3.4.2) [24].

## 3. Results

**Figure 1:**
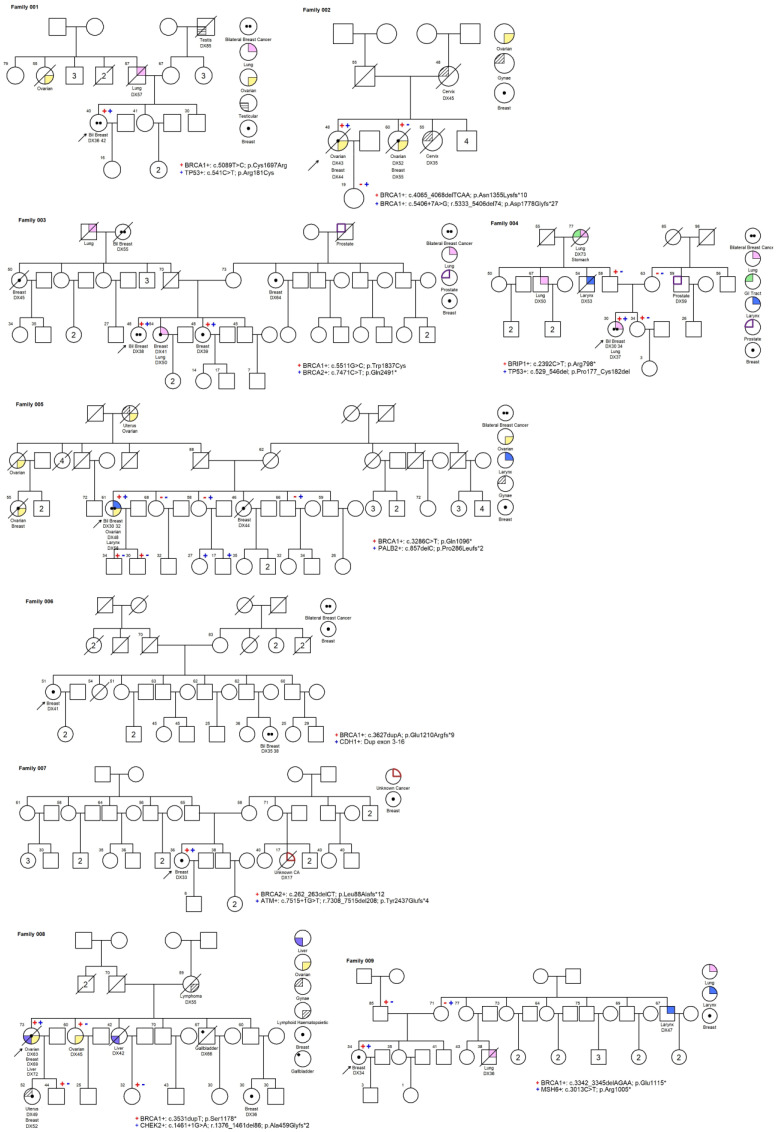
The pedigrees of individuals from family 1 to family 9 who carried double mutations. Symbols are used as follows: male—square; female—circle; marriage—square and circle linked by a horizontal line; cancer affected individuals—refer to legends; proband—pointed with an arrow; mutation status—“+” tested mutation carrier and “-“ tested non-carrier; deceased—circle or square with a line running through it; Roman numerals symbolize number of siblings of same gender. * only including 1st and 2nd degree.

In this cohort of 3649 high-risk Chinese breast cancer patients, pathogenic or likely pathogenic (P/LP) DH germline mutations were identified in 9 (0.25%) Chinese probands with breast cancer. These mutations involved *ATM*, *BRCA1*, *BRCA2*, *BRIP1*, *CDH1*, *CHEK2*, *MSH6*, *PALB2*, and *TP53* genes (Table 1). Seven out of nine (77.8%) DH carriers carried at least one P/LP *BRCA1* mutation variant. The mean and median ages of first breast cancer diagnosis were 39.8 and 36, respectively, ranging from 30 to 69 years. Out of the nine DH carriers, a total of four (44.4%) had bilateral breast cancer, and four out of these nine subjects (44.4%) had other cancers in addition to the breast cancer. Three patients had breast and ovarian cancers, and two of them had an additional third or fourth primary cancers of the larynx and liver. Among the DH carriers, 44.4% had a family history of breast or ovarian cancer, while only 22.2% had a family history of prostate cancer. The majority of the breast cancers were invasive ductal carcinoma (IDC) (92.3%), and 54.5% of them were high-grade. Around 55% of the cases were ER/PR+, while only 27% were HER2+. Overall, 40% of cases were triple-negative breast cancers. Among seven families, 19 family members were available for the genetic test, and only one family had more than one DH carrier. This was the sister of a proband in family 003 who had personally diagnosed breast cancer at 39. In the same generation, three out of four siblings, who did not come for genetic test, had their breast cancers diagnosed at a young age (<45). The pedigrees and detailed surveillance managements of these families are detailed in Figure 1 and the Supplementary Materials. There were no consanguineous families in our cohort.

**Figure 2:**
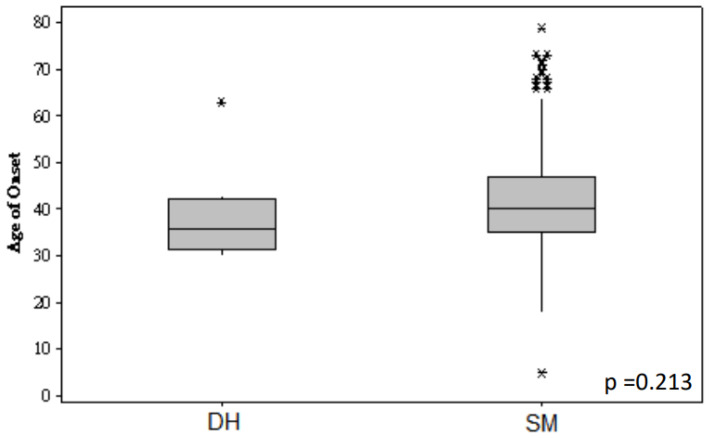
Boxplot showing the age of onset of cancer among patients carrying double heterozygous (DH) mutations and simple mutations (SM). * represented the extreme values in the data set.

In this cohort of 3649 high-risk Chinese breast cancer patients, 511 probands were found to have P/LP germline simple heterozygote (SH) mutations from our HBOC-related gene panel. The age of diagnosis, personal history of multiple cancers, bilateral breast cancer, family history of cancers, and tumor characteristics were compared between DH mutation carriers and SH mutation carriers. In this small collection of DH individuals, the ages of first cancer diagnosis (*p* = 0.213) (Figure 2) and all other clinicopathologic characteristics were similar between DH and DM carriers (Table 2). DH carriers did not show worse phenotypes or more significantly downhill clinical presentations than SH mutation carriers. Patients with DH mutations had a greater family history of ovarian cancers than SH mutation carriers (*p* = 0.031), but there was no significant difference between DH and SH mutation carriers on those with a family history of breast and prostate cancer.

## 4. Discussion

With the availability and popularity of next-generation sequencing (NGS), genetic testing has become widely used in clinical laboratory settings. Sequencing of multiple-gene panels or even whole exomes/genomes leads to the rapid expansion of data on the identified mutations. More worldwide-coverage studies, not limited to specific populations such as Ashkenazi Jews, have reported on breast cancer patients with DH mutations (see Supplementary Table S1). Among the DH mutations identified from a mixed population reported by CIMBA, the most common DH mutations from the *BRCA* genes involved the inheritance of two of the three common Jewish mutations: 5.4% of women inherited *BRCA1*: c.5266dup; p.(Gln1756fs) and *BRCA2*: c.5946del; p.(Ser1982fs), and 33.3% of women inherited *BRCA1*: c.68_69del; p.(Glu23fs) and *BRCA2*: c.5946del; p.(Ser1982fs) [20]. None of these Jewish founder mutations have been found in our Chinese DH cohort. Our group and others have reported a total of 10 DH mutations in *BRCA1* and *BRCA2*, but not other HBOC genes, in Asians. Two Asians from the US had *BRCA1*: c.1016del; p.(Lys339fs) and *BRCA1*: c.5136G>A; p.(Trp1712Ter) and *BRCA2*: c.7379_7382del; p.(Asn2460fs) and *BRCA2*: c.4965del; p.(Cys1654_Tyr1655insTer) [20]. One Japanese DH proband had *BRCA1*: c.188T>A; p.(Leu63Ter) and *BRCA2*: c.5576_5579del; p.(Ile1859fs) mutations [19]. Five Koreans with breast cancers have also been reported to have had DH *BRCA* mutations, including *BRCA1*: c.1504_1508del; p.(Leu502fs), c.3627dup; p.(Glu1210fs), c.390C>A; p.(Tyr130Ter), c.4981G>T; p.(Glu1661Ter), and c.5030_5033del; p.(Thr1677fs) and *BRCA2*: c.2798_2799del; p.(Thr933fs), c.6724_6725del; p.(Asp2242fs), c.3018del; p.(Gly1007fs), c.5946_5949del; p.(Ser1982fs), and c.1399A>T; p.(Lys467Ter) [17,18]. In our Chinese cohort, we identified two *BRCA* DH cases; one of them carried c.5511G>C; p.(Trp1837Cys) in the *BRCA1* gene and c.7471C>T; p.(Gln2491*) in the *BRCA2* gene, and another carrier had double *BRCA1* mutations of c.4065_4068delTCAA; p.(Asn1355Lysfs*10) and c.5406+7A>G; r.5333_5406del74; p.(Asp1778Glyfs*27) [25]. In the Asian populations, many of these DH mutations were observed just once, suggesting no evidence of founder effects. We anticipate a better understanding of the nature of DH among Asians as genetic data of the Asian breast cancer patients accumulates.

A previous study on a Jewish population reported no significant differences in breast cancer occurrence between DH and SH carriers: 46.7% of DH women had a personal history of breast carcinoma, while 40.2% of carriers of a single mutation had such a personal history (*p* = 0.801) [26]. Another study compared the age at breast cancer diagnosis with DH and SH in the *BRCA1* and *BRCA2* genes. The ages were similar regarding to DH versus SH in the *BRCA1* gene (*p* = 0.231), but the age of diagnosis was, on average, 4.5 years earlier for DH individuals than SH patients in the *BRCA2* gene (*p* < 0.001) [20]. This study also demonstrated that patients with two inherited pathogenic mutations were more likely to develop ovarian cancer than those carrying a *BRCA2* pathogenic variant alone [20]. In our small cohort, our patients with DH and SH mutations showed that the DH mutation carriers also had stronger family histories of ovarian cancer than SH mutation carriers (*p* = 0.031); however, the relationship between having ovarian cancer and DH may not be that simple, as all three of our DH carriers with both breast and ovarian cancers carried the *BRCA1* mutation instead of the *BRCA2* mutation. In our registry, patients with both breast and ovarian cancers were more likely to carry a *BRCA1* mutation (24.7%) than a *BRCA2* mutation (10.8%) [27]. The higher incidence of developing a personal and family history of ovarian cancer may not be solely related to DH, as there were modifiers such as a Chinese population trend or bias within the cohort in our registry.

A study of eight DH individuals suggested that DH individuals develop breast cancer at an earlier age and have more severe disease than those with SH in the *BRCA* gene [28]. Two studies on DH in the *CHEK2* gene or the *ATM* gene with *BRCA* mutations demonstrated that the presence of second gene defects did not further increase the risk of disease in those patients who already carried SH in the *BRCA* genes [16,29]. On the other hand, another report on 17 DH breast cancer patients stemming from Russia, Belarus, and Poland showed no association with an earlier age at diagnosis for the patients with DH. These Eastern European families did not have a stronger family history than the SH families [12]. A single *BRCA1*/*PALB2* DH case showed no associations with the severity of the phenotype compared to SH carriers [30]. In our cohort, there was no significant difference in these clinicopathological parameters between DH and SH carriers (Table 2).

Aggregated data showed bilateral breast cancers were not particularly prevalent in non-Asian DH carriers. Among a total of 7/81 (8.6%) non-Asian DH patients with bilateral breast cancers reported in the literatures, including Australian, Caucasian, French Canadian, Hispanic, Italian, Scottish, and Slavic individuals, the frequency of bilateral breast cancers was only 10.8% (7/65, see Supplementary Table S1). However, bilateral breast cancers were observed in 44.4% (4/9) of patients in our cohort, suggesting Chinese with DH may have a higher chance of having bilateral breast cancer than other populations (*p* = 0.0237). For our locality, we suggest conducting more frequent surveillance imaging or taking aggressive prophylactic measures for patients and family members who are DH.

In conclusion, the clinical oncological features of DH individuals might be ethnically specific. Carriers of DH mutations were not noticeably different from SH carriers with respect to an earlier onset and a worsening phenotype. For the Chinese population in our cohort of high-risk referral patients of the Hong Kong Hereditary Breast Cancer Family Registry, DH was associated with a higher prevalence of bilateral breast cancers. Chinese individuals with DH may have a higher chance of having bilateral breast cancer than other populations. Although the validity of this observation might need to be confirmed using a larger cohort, and though it might be premature at this stage to recommend a general management strategy for all DH families, tight surveillance and possibly more-aggressive treatment plans could be tailored to specific family needs in our locality.

## 5. Conclusions

We conducted a review of double heterozygous (DH) carriers among a high-risk breast cancer cohort in Hong Kong and evaluated whether DH carriers exhibited a worse phenotype or a significantly deteriorating clinical presentation compared to single heterozygous (SH)-mutation carriers. However, we found that DH carriers did not show a worse phenotype or a significantly deteriorating clinical presentation when compared to SH mutation carriers. Based on the review of DH cases available in the literature, it appears that Chinese individuals with DH mutations may have a higher chance of developing bilateral breast cancer compared to other populations. However, whether it is worth recommending a more stringent surveillance program and a more aggressive treatment plan for DH families may require confirmation using a larger cohort.

## Figures and Tables

**Table 1:** Characteristics of DH carriers.

Patient ID	Gene	Pathogenic Variants	VAF	Personal Cancer (Age of Diagnosis)	Metastasis or Relapse	Breast (Histology/ ER/PR/HER2)	Family Cancer History * (Age of Diagnosis)
001	BRCA1 TP53	c.5089T>C; p.Cys1697Arg c.541C>T; p.Arg181Cys	37.1 55.1	Bilateral Breast (36 42)	N/N	IDC/+/+/− IDC/+/+/−	Lung (57) Ovarian (UK) Testis (85)
002	BRCA1 BRCA1	c.4065_4068delTCAA; p.Asn1355Lysfs * 10 c.5406+7A>G; r.5333_5406del74; p.Asp1778Glyfs * 27	54.1 43.2	Breast (44) Ovarian (43)	N/Y	IDC/−/−/−	Ovarian and Breast (52 55) Cervical (35) Cervical (45)
003	BRCA1 BRCA2	c.5511G>C; p.Trp1837Cys c.7471C>T; p.Gln2491 *	Via Sanger sequencing	Bilateral Breast (38)	N/N	IDC/−/−/− DCIS/+/−/−	Breast (39) Breast and Lung (41 50) Breast (45) Breast (64) Bilateral Breast (55) Prostate (UK) Lung (UK)
004	BRIP1 TP53	c.2392C>T; p.Arg798 * c.529_546del; p.Pro177_Cys182del	58.0 33.1	Bilateral Breast (30 34) Lung (37)	N/N	IDC/+/+/+ IDC/+/+/+	Prostate (59) Larynx (53) Lung (50) Lung and Stomach (73 UK)
005	BRCA1 PALB2	c.3286C>T; p.Gln1096 * c.857delC; p.Pro286Leufs * 2	47.4 48.7	Bilateral Breast (30 32) Ovarian (48) Laryngeal (58)	N/N	IDC/−/UK/UK IDC/−/UK/UK	Bilateral Breast (35 38)
006	BRCA1 CDH1	c.3627dupA; p.Glu1210Argfs * 9 Dup exon 3–16	46.5 NA	Breast (41)	N/N	IDC/−/−/−	Breast (44) Ovarian (UK) Uterus and Ovarian (UK)
007	ATM BRCA2	c.7515+1G>T; r.7308_7515del208; p.Tyr2437Glufs * 4 c.262_263delCT; p.Leu88Alafs * 12	50.2 37.7	Breast (33)	N/N	IDC/+/+/−	Cancer of unknown primary (17)
008	BRCA1 CHEK2	c.3531dupT; p.Ser1178 * c.1461+1G>A; r.1376_1461del86; p.Ala459Glyfs * 2	48.9 40.2	Ovarian (63) Breast (69) Liver (72)	N/N	IDC/+/+/+	Breast and Ovarian (49) Ovarian (45) Lymphoma (42) Gallbladder (66) Lymphoma (55) Breast (36)
009	BRCA1 MSH6	c.3342_3345del; p.Glu1115 * c.3013C>T; p.Arg1005 *	51.3 52.0	Breast (34)	N/N	IDC/−/−/−	Larynx (47)

* only including 1st and 2nd degree.

**Table 2:** Characteristics of double heterozygous (DH) mutation and simple heterozygous mutation (SH) carriers.

Patients Characteristics	Double Mutations (N = 9)	Single Mutation (N = 511)	Total (N = 520)	*p*-Value
Diagnosis age				
Median	36	41	40.5	0.213
Range	30–69	18–79	18–79	
Multiple personal cancers				
Yes	4 (44.4%)	97 (19.0%)	100 (19.2%)	0.076
No	5 (55.6%)	414 (81.0%)	420 (80.%)	
1st- or 2nd-degree family history of breast cancer				
Yes	4 (44.4%)	295 (57.7%)	299 (57.5%)	0.505
No	5 (55.6%)	216 (42.3%)	221 (42.5%)	
1st- or 2nd-degree family history of ovarian cancer				
Yes	4 (44.4%)	73 (14.3%)	77 (14.8%)	0.031
No	5 (55.6%)	438 (85.7%)	443 (85.2%)	
1st- or 2nd-degree family history of prostate cancer				
Yes	2 (22.2%)	45 (8.8%)	47 (9.0%)	0.192
No	7 (77.8%)	466 (91.2%)	473 (91.0%)	
Bilateral breast cancer				
Yes	4 (44.4%)	148 (29.0%)	152 (29.2%)	0.295
No	5 (55.6%)	363 (71.0%)	368 (70.8%)	
**Tumor Characteristics**	**Double Mutations N = 13**	**Single Mutation** **N = 659**	**Total** **N = 672**	***p*-Value**
Stage				
0	1 (8.3%)	89 (14.2%)	90 (14.1%)	0.897
1	5 (41.7%)	233 (37.2%)	238 (37.3%)	
2	4 (33.3%)	224 (35.7%)	228 (35.7%)	
3	2 (16.7%)	61 (9.7%)	63 (9.9%)	
4	0 (0%)	20 (3.2%)	20 (3.1%)	
Not stated	1	32	33	
Histology				
ductal	12 (92.3%)	492 (77.2%)	504 (77.5%)	0.689
in-situ	1 (7.7%)	81 (12.7%)	82 (12.6%)	
other	0 (0%)	64 (10.1%)	64 (9.9%)	
Not stated	0	22	22	
Invasive grade				
1	1 (10.0%)	25 (5.4%)	26 (5.5%)	0.459
2	3 (30.0%)	189 (40.7)	192 (40.4%)	
3	6 (60.0%)	251 (54.0)	257 (54.1%)	
Not stated	2	113	115	
ER				
Neg	6 (46.2%)	219 (36.6%)	225 (36.8%)	0.564
Pos	7 (53.9%)	379 (63.4%)	386 (63.2%)	
Not stated	0	61	61	
PR				
Neg	5 (45.5%)	285 (48.8%)	290 (48.7%)	1.000
Pos	6 (54.6%)	299 (51.2%)	305 (51.3%)	
Not stated	2	75	77	
Her2				
Neg	8 (72.7%)	432 (77.3%)	440 (77.2%)	0.515
Equivocal	0 (0%)	44 (7.9%)	44 (7.7%)	
Pos	3 (27.3%)	83 (14.9%)	86 (15.1%)	
Not stated	2	100	102	
TNBC				
Yes	4 (40.0%)	157 (30.0%)	372 (69.8%)	0.499
No	6 (60.0%)	366 (70.0%)	161 (30.2%)	
Not stated	2	51	57	

## Data Availability

The dataset supporting the conclusions of this article is included within the article and its Supplementary Materials.

## Supplementary Materials

The following supporting information can be downloaded at https://www.mdpi.com/article/10.3390/cancers16142547/s1. Table S1: Summary of reported double heterozygosity germline mutations; Supplementary text: Surveillance management and family study for the families with DH mutations. References [31,32,33,34,35,36,37,38,39,40,41,42,43,44,45] are cited in Supplementary Materials.

## References

[1] Chen S., Iversen E.S., Friebel T., Finkelstein D., Weber B.L., Eisen A., Peterson L.E., Schildkraut J.M., Isaacs C., Peshkin B.N. (2006). Characterization of BRCA1 and BRCA2 mutations in a large United States sample. J. Clin. Oncol..

[2] Tai Y.C., Domchek S., Parmigiani G., Chen S. (2007). Breast cancer risk among male BRCA1 and BRCA2 mutation carriers. J. Natl. Cancer Inst..

[3] Frank T.S., Deffenbaugh A.M., Reid J.E., Hulick M., Ward B.E., Lingenfelter B., Gumpper K.L., Scholl T., Tavtigian S.V., Pruss D.R. (2002). Clinical characteristics of individuals with germline mutations in BRCA1 and BRCA2: Analysis of 10,000 individuals. J. Clin. Oncol..

[4] Ramus S.J., Friedman L.S., Gayther S.A., Ponder B.A., Bobrow L., van der Looji M., Papp J., Olah E. (1997). A breast/ovarian cancer patient with germline mutations in both BRCA1 and BRCA2. Nat. Genet..

[5] Friedman E., Bar-Sade Bruchim R., Kruglikova A., Risel S., Levy-Lahad E., Halle D., Bar-On E., Gershoni-Baruch R., Dagan E., Kepten I. (1998). Double heterozygotes for the Ashkenazi founder mutations in BRCA1 and BRCA2 genes. Am. J. Hum. Genet..

[6] Randall T.C., Bell K.A., Rebane B.A., Rubin S.C., Boyd J. (1998). Germline mutations of the BRCA1 and BRCA2 genes in a breast and ovarian cancer patient. Gynecol. Oncol..

[7] Moslehi R., Russo D., Phelan C., Jack E., Antman K., Narod S. (2000). An unaffected individual from a breast/ovarian cancer family with germline mutations in both BRCA1 and BRCA2. Clin. Genet..

[8] Bell D.W., Erban J., Sgroi D.C., Haber D.A. (2002). Selective loss of heterozygosity in multiple breast cancers from a carrier of mutations in both BRCA1 and BRCA2. Cancer Res..

[9] Leegte B., van der Hout A.H., Deffenbaugh A.M., Bakker M.K., Mulder I.M., ten Berge A., Leenders E.P., Wesseling J., de Hullu J., Hoogerbrugge N. (2005). Phenotypic expression of double heterozygosity for BRCA1 and BRCA2 germline mutations. J. Med. Genet..

[10] Bogdanova N.V., Antonenkova N.N., Rogov Y.I., Karstens J.H., Hillemanns P., Dörk T. (2010). High frequency and allele-specific differences of BRCA1 founder mutations in breast cancer and ovarian cancer patients from Belarus. Clin. Genet..

[11] Sokolenko A.P., Iyevleva A.G., Mitiushkina N.V., Suspitsin E.N., Preobrazhenskaya E.V., Kuligina E.S.h., Voskresenskiy D.A., Lobeiko O.S., Krylova N.Y., Gorodnova T.V. (2010). Hereditary breast-ovarian cancer syndrome in Russia. Acta Naturae.

[12] Sokolenko A.P., Bogdanova N., Kluzniak W., Preobrazhenskaya E.V., Kuligina E.S., Iyevleva A.G., Aleksakhina S.N., Mitiushkina N.V., Gorodnova T.V., Bessonov A.A. (2014). Double heterozygotes among breast cancer patients analyzed for BRCA1, CHEK2, ATM, NBN/NBS1, and BLM germ-line mutations. Breast Cancer Res. Treat..

[13] Chekmariova E.V., Sokolenko A.P., Buslov K.G., Iyevleva A.G., Ulibina Y.M., Rozanov M.E., Mitiushkina N.V., Togo A.V., Matsko D.E., Voskresenskiy D.A. (2006). CHEK2 1100delC mutation is frequent among Russian breast cancer patients. Breast Cancer Res. Treat..

[14] Bogdanova N., Cybulski C., Bermisheva M., Datsyuk I., Yamini P., Hillemanns P., Antonenkova N.N., Khusnutdinova E., Lubinski J., Dörk T. (2009). A nonsense mutation (E1978X) in the ATM gene is associated with breast cancer. Breast Cancer Res. Treat..

[15] Megid T.B.C., Barros-Filho M.C., Pisani J.P., Achatz M.I. (2022). Double heterozygous pathogenic variants prevalence in a cohort of patients with hereditary breast cancer. Front. Oncol..

[16] Heidemann S., Fischer C., Engel C., Fischer B., Harder L., Schlegelberger B., Niederacher D., Goecke T.O., Doelken S.C., Dikow N. (2012). Double heterozygosity for mutations in BRCA1 and BRCA2 in German breast cancer patients: Implications on test strategies and clinical management. Breast Cancer Res. Treat..

[17] Noh J.M., Choi D.H., Nam S.J., Lee J.E., Kim J.W., Kim S.W., Kang E., Lee M.H., Ahn S.H., Kim K.S. (2012). Characteristics of double heterozygosity for BRCA1 and BRCA2 germline mutations in Korean breast cancer patients. Breast Cancer Res. Treat..

[18] Choi D.H., Lee M.H., Bale A.E., Carter D., Haffty B.G. (2004). Incidence of BRCA1 and BRCA2 mutations in young Korean breast cancer patients. J. Clin. Oncol..

[19] Nomizu T., Matsuzaki M., Katagata N., Kobayashi Y., Sakuma T., Monma T., Saito M., Watanabe F., Midorikawa S., Yamaguchi Y. (2015). A case of familial breast cancer with double heterozygosity for BRCA1 and BRCA2 genes. Breast Cancer.

[20] Rebbeck T.R., Friebel T.M., Mitra N., Wan F., Chen S., Andrulis I.L., Apostolou P., Arnold N., Arun B.K., Barrowdale D. (2016). Inheritance of deleterious mutations at both BRCA1 and BRCA2 in an international sample of 32,295 women. Breast Cancer Res..

[21] Kwong A., Shin V.Y., Au C.H., Law F.B., Ho D.N., Ip B.K., Wong A.T., Lau S.S., To R.M., Choy G. (2016). Detection of Germline Mutation in Hereditary Breast and/or Ovarian Cancers by Next-Generation Sequencing on a Four-Gene Panel. J. Mol. Diagn..

[22] Neben C.L., Zimmer A.D., Stedden W., van den Akker J., O’Connor R., Chan R.C., Chen E., Tan Z., Leon A., Ji J. (2019). Multi-Gene Panel Testing of 23,179 Individuals for Hereditary Cancer Risk Identifies Pathogenic Variant Carriers Missed by Current Genetic Testing Guidelines. J. Mol. Diagn..

[23] Auton A., Brooks L.D., Durbin R.M., Garrison E.P., Kang H.M., Korbel J.O., Marchini J.L., McCarthy S., McVean G.A., 1000 Genomes Project Consortium (2015). A global reference for human genetic variation. Nature.

[24] R Core Team (2019). R: A Language and Environment for Statistical Computing.

[25] Kwong A., Ho C.Y.S., Shin V.Y., Au C.H., Chan T.L., Ma E.S.K. (2021). A Case Report of Germline Compound Heterozygous Mutations in the *BRCA1* Gene of an Ovarian and Breast Cancer Patient. Int. J. Mol. Sci..

[26] Lavie O., Narod S., Lejbkowicz F., Dishon S., Goldberg Y., Gemer O., Rennert G. (2011). Double heterozygosity in the BRCA1 and BRCA2 genes in the Jewish population. Ann. Oncol..

[27] Kwong A., Ho C.Y.S., Shin V.Y., Au C.H., Luk W.P., Fung L.H., Chan T.L., Chan K.K.L., Ngan H.Y.S., Ma E.S.K. (2022). Germline mutations in Chinese ovarian cancer with or without breast cancer. Mol. Genet. Genom. Med..

[28] Meijers-Heijboer H., van den Ouweland A., Klijn J., Wasielewski M., de Snoo A., Oldenburg R., Hollestelle A., Houben M., Crepin E., van Veghel-Plandsoen M. (2002). Low-penetrance susceptibility to breast cancer due to CHEK2(*)1100delC in noncarriers of BRCA1 or BRCA2 mutations. Nat. Genet..

[29] Turnbull C., Seal S., Renwick A., Warren-Perry M., Hughes D., Elliott A., Pernet D., Peock S., Adlard J.W., Barwell J. (2012). Gene-gene interactions in breast cancer susceptibility. Hum. Mol. Genet..

[30] Pern F., Bogdanova N., Schürmann P., Lin M., Ay A., Länger F., Hillemanns P., Christiansen H., Park-Simon T.W., Dörk T. (2012). Mutation analysis of BRCA1, BRCA2, PALB2 and BRD7 in a hospital-based series of German patients with triple-negative breast cancer. PLoS ONE..

[31] Keupp K., Hampp S., Hübbel A., Maringa M., Kostezka S., Rhiem K., Waha A., Wappenschmidt B., Pujol R., Surrallés J. (2019). Biallelic germline BRCA1 mutations in a patient with early onset breast cancer, mild Fanconi anemia-like phenotype, and no chromosome fragility. Mol. Genet. Genom. Med..

[32] Sukumar J., Kassem M., Agnese D., Pilarski R., Ramaswamy B., Sweet K., Sardesai S. (2021). Concurrent germline BRCA1, BRCA2, and CHEK2 pathogenic variants in hereditary breast cancer: A case series. Breast Cancer Res. Treat..

[33] Meynard G., Mansi L., Lebahar P., Villanueva C., Klajer E., Calcagno F., Vivalta A., Chaix M., Collonge-Rame M.A., Populaire C. (2017). First description of a double heterozygosity for BRCA1 and BRCA2 pathogenic variants in a French metastatic breast cancer patient: A case report. Oncol. Rep..

[34] Palmirotta R., Lovero D., Stucci L.S., Silvestris E., Quaresmini D., Cardascia A., Silvestris F. (2018). Double Heterozygosity for BRCA1 Pathogenic Variant and BRCA2 Polymorphic Stop Codon K3326X: A Case Report in a Southern Italian Family. Int. J. Mol. Sci..

[35] Zuradelli M., Peissel B., Manoukian S., Zaffaroni D., Barile M., Pensotti V., Cavallari U., Masci G., Mariette F., Benski A.C. (2010). Four new cases of double heterozygosity for BRCA1 and BRCA2 gene mutations: Clinical, pathological, and family characteristics. Breast Cancer Res. Treat..

[36] Liede A., Rehal P., Vesprini D., Jack E., Abrahamson J., Narod S.A. (1998). A breast cancer patient of Scottish descent with germ-line mutations in BRCA1 and BRCA2. Am. J. Hum. Genet..

[37] Tesoriero A., Andersen C., Southey M., Somers G., McKay M., Armes J., McCredie M., Giles G., Hopper J.L., Venter D. (1999). De novo BRCA1 mutation in a patient with breast cancer and an inherited BRCA2 mutation. Am. J. Hum. Genet..

[38] Le Page C., Rahimi K., Rodrigues M., Heinzelmann-Schwarz V., Recio N., Tommasi S., Bataillon G., Portelance L., Golmard L., Meunier L. (2020). Clinicopathological features of women with epithelial ovarian cancer and double heterozygosity for BRCA1 and BRCA2: A systematic review and case report analysis. Gynecol. Oncol..

[39] Pilato B., De Summa S., Danza K., Lambo R., Paradiso A., Tommasi S. (2010). Maternal and paternal lineage double heterozygosity alteration in familial breast cancer: A first case report. Breast Cancer Res. Treat..

[40] Pedroni M., Di Gregorio C., Cortesi L., Reggiani Bonetti L., Magnani G., Simone M.L., Medici V., Priore Oliva C., Marino M., Ponz de Leon M. (2024). Double heterozygosity for BRCA1 and hMLH1 gene mutations in a 46-year-old woman with five primary tumors. Tech. Coloproctol..

[41] Bell K., Hodgson N., Levine M., Sadikovic B., Zbuk K. (2014). Double heterozygosity for germline mutations in BRCA1 and p53 in a woman with early onset breast cancer. Breast Cancer Res. Treat..

[42] Ataei-Kachouei M., Nadaf J., Akbari M.T., Atri M., Majewski J., Riazalhosseini Y., Garshasbi M. (2015). Double Heterozygosity of BRCA2 and STK11 in Familial Breast Cancer Detected by Exome Sequencing. Iran. J. Public Health.

[43] Thiffault I., Hamel N., Pal T., McVety S., Marcus V.A., Farber D., Cowie S., Deschênes J., Meschino W., Odefrey F. (2004). Germline truncating mutations in both MSH2 and BRCA2 in a single kindred. Br. J. Cancer.

[44] Cote S., Arcand S.L., Royer R., Nolet S., Mes-Masson A.M., Ghadirian P., Foulkes W.D., Tischkowitz M., Narod S.A., Provencher D. (2012). The BRCA2 c.9004G>A (E2002K) [corrected] variant is likely pathogenic and recurs in breast and/or ovarian cancer families of French Canadian descent. Breast Cancer Res. Treat..

[45] Vietri M.T., Caliendo G., D’Elia G., Resse M., Casamassimi A., Minucci P.B., Dello Ioio C., Cioffi M., Molinari A.M. (2020). Five Italian Families with Two Mutations in *BRCA* Genes. Genes.

