# Supplementary material for: Double Heterozygosity for Germline Mutations in Chinese Breast Cancer Patients
Source: Cancers (Basel). 2024 Jul 15;16(14):2547. doi: 10.3390/cancers16142547 (PMC11274758; doi:10.3390/cancers16142547)
Supplement: Supplementary file 1 [file cancers-16-02547-s001.zip › cancers-3068792-supplementary.pdf]

Supplementary Table S1. Summary of reported double heterozygosity germline mutations.

Genes	Race	Country	Dx	Personal Cancer(s)	FH	Mutations	Reference
Biallelic *BRCA1*	Caucasian	Germany	30	Breast Cancer	Maternal Grandfather: Prostate Cancer<br>Paternal Grandmother: Breast Cancer	*BRCA1*: c.181T>G; p.Cys61Gly<br>*BRCA1*: c.5096G>A; p.Arg1699Gln	31
Biallelic *BRCA2*	Italian	Italy	45	Breast Cancer	Mother: Breast Cancer<br>Mother's Brother: Male Breast Cancer<br>Maternal Cousin: Breast Cancer	*BRCA2*: c.631G>A; p.Val211Ile<br>*BRCA2*: c.7008-2A>T (IVS13-2A>T)	45
Biallelic *BRCA2*	Italian	Italy	65	Breast Cancer	Brother: Colon Cancer<br>Nieces x6: Breast Cancers	*BRCA2*: c.631G>A; p.Val211Ile<br>*BRCA2*: c.7008-2A>T (IVS13-2A>T)	45
Biallelic *BRCA2*	Italian	Italy	60	Breast Cancer	Sister: Breast Cancer<br>Brother: Leukemia<br>Father: Pancreatic Cancer<br>Paternal Cousin x4: Breast Cancer<br>Nephew: Breast Cancer	*BRCA2*: c.631G>A; p.Val211Ile<br>*BRCA2*: c.7008-2A>T (IVS13-2A>T)	45
BRCA1/ATM	Slavic	Belarus	40	Breast Cancer	No Cancer	*BRCA1*: c.181T>G; p.Cys61Gly<br>*ATM*: c.5932G>T; p.Glu1978Ter	12
BRCA1/ATM	Slavic	Belarus	42	Breast Cancer	No Cancer	*BRCA1*: c.181T>G; p.Cys61Gly<br>*ATM*: c.5932G>T; p.Glu1978Ter	12
BRCA1/BLM	Slavic	Russia	68<br>50	Breast Cancer<br>Ovarian Cancer	Sister: Breast Cancer	*BRCA1*: c.5266dup; p.Gln1756fs<br>*BLM*: c.1642C>T; p.Gln548Ter	12
BRCA1/BLM	Slavic	Russia	67<br>58	Breast Cancer<br>Lymphoma	Mother: Gastric Cancer<br>Father: Lung Cancer<br>Paternal aunt: Breast Cancer	*BRCA1*: c.5266dup; p.Gln1756fs<br>*BLM*: c.1642C>T; p.Gln548Ter	12
BRCA1/BLM	Slavic	Belarus	44	Breast Cancer	Mother: Breast Cancer<br>Sister: Breast Cancer<br>Paternal aunt: Breast Cancer	*BRCA1*: c.5266dup; p.Gln1756fs<br>*BLM*: c.1642C>T; p.Gln548Ter	12

BRCA1/BLM	Slavic	Belarus	45	Breast Cancer	Sister: Breast Cancer<br>Father: Gastric Cancer	*BRCA1*: c.5266dup; p.Gln1756fs<br>*BLM*: c.1642C>T; p.Gln548Ter	12
BRCA1/CHEK2	Slavic	Belarus	58	Breast Cancer	No Cancer	*BRCA1*: c.181T>G; p.Cys61Gly<br>*CHEK2*: c.1100del; p.Thr367fs	12
BRCA1/CHEK2	Slavic	Poland	54	Breast Cancer	Sister: Breast Cancer<br>Grandmother: Breast Cancer	*BRCA1*: c.5266dup; p.Gln1756fs<br>*CHEK2*: c.444+1G>A	12
BRCA1/CHEK2	Slavic	Russia	52	Breast Cancer	Mother: Breast Cancer	*BRCA1*: c.5266dup; p.Gln1756fs<br>*CHEK2*: Del exon 9-10;<br>NC_000022.10:g.27416941_27422508del	12
BRCA1/CHEK2	Slavic	Russia	42	Breast Cancer	Sister: Ovarian Cancer	*BRCA1*: c.5266dup; p.Gln1756fs<br>*CHEK2*: Del exon 9-10;<br>NC_000022.10:g.27416941_27422508del	12
BRCA1/BRCA2/ CHEK2	Caucasian	USA	55	Bilateral Breast Cancer	Mother: Bilateral Breast Cancer<br>Sister: Breast Cancer<br>Maternal Grandmother: Breast Cancer<br>Brother: Melanoma<br>Daughter: Breast Cancer<br>Daughter: Kartagener's Syndrome	*BRCA1*: c.181T>G; p.Cys61Gly<br>*BRCA2*: c.4398_4402del; p.Leu1466fs<br>*CHEK2*: c.1100del; p.Thr367fs	32
BRCA1/BRCA2	Caucasian	France	46	Bilateral Breast Cancer	Paternal Grandmother: Leukemia<br>Paternal Grandfather: Esophagus Cancer<br>Father's Half-Brother: Pancreatic Cancer<br>Maternal Grandmother: Breast Cancer<br>Maternal Grandfather: CNS Cancer<br>Maternal Aunt: Bilateral Breast Cancer<br>Maternal Aunt: Breast Cancer	*BRCA1*: c.1016dupA; p.Val340fs<br>*BRCA2*: c.6814delA; p.Arg2272fs	33
BRCA1/BRCA2	Asian	USA	NI	Breast Cancer	FH not informative	*BRCA1*: c.1016delA; p.Val340fs<br>*BRCA2*: c.7379_7382del; p.Asn2460fs	20

BRCA1/*BRCA2*	Hispanic	USA	NI	Breast Cancer	FH not informative	*BRCA1*: c.1390del; p.Thr464fs<br>*BRCA2*: c.658_659del; p.Val220fs	20
BRCA1/*BRCA2*	Korean	Korea	26	Breast Cancer	Grandmother: Stomach Cancer	*BRCA1*: c.1504_1508del; p.Leu502fs<br>*BRCA2*: c.2798_2799del; p.Thr933fs	18
BRCA1/*BRCA2*	Italian	Italy	40<br>54	Bilateral Breast Cancer<br>Melanoma	Sister: Breast Cancer<br>Maternal Grandmother: Breast Cancer<br>Maternal Aunt: Ovarian Cancer<br>Maternal Cousin: Pancreatic Cancer<br>Maternal Cousin: Melanoma<br>Paternal Uncle: Intestinal Cancer	*BRCA1*: c.1687C>T; p.Gln563Ter<br>*BRCA2*: c.9976A>T; p.Lys3326Ter	34
BRCA1/*BRCA2*	Italian	Italy	56<br>58	Breast Cancer<br>Ovarian Cancer	Brother: Gastric Cancer<br>Father: Leukemia<br>Paternal Aunt: Breast Cancer<br>Mother: Breast Cancer<br>Maternal Grandmother: Gastric Cancer<br>Maternal Aunt: Breast Cancer<br>Maternal Uncle: Colon Cancer<br>Maternal Cousin: Colon Cancer<br>Maternal Cousin: Cancer of Unknown Origin	*BRCA1*: c.1687C>T; p.Gln563Ter<br>*BRCA2*: c.6469C>T; p.Gln2157Ter	35
BRCA1/*BRCA2*	Italian	Italy	49	Bilateral Breast Cancer	Paternal Cousin: Breast and Ovarian Cancer<br>Paternal Cousin: Prostate Cancer<br>Father's Brother: Laryngeal Cancer<br>Father's Brother: Bladder Cancer<br>Father's Sister: Breast and Ovarian Cancer<br>Father's Sister: Ovarian Cancer<br>Father's Sister: Breast Cancer<br>Father's Sister: Colon Cancer	*BRCA1*: c.547+2T>A (IVS8+2T>A)<br>*BRCA2*: c.2830A>T; p.Lys944Ter	45

BRCA1/*BRCA2*	Caucasian	Austria	NI	Breast Cancer	FH not informative	*BRCA1*: c.181T>G; p.Cys61Gly<br>*BRCA2*: c.1318_1319dup; p.Thr441fs	20
BRCA1/*BRCA2*	Japanese	Japan	59	Breast Cancer	Sister: Bilateral Breast Cancer<br>Brother: Stomach Cancer<br>Brother: Rectal Cancer<br>Mother: Ovarian Cancer<br>Paternal Aunt: Ovarian Cancer<br>Paternal Aunt: Ovarian Cancer<br>Paternal Cousin: Breast & Endometrial Cancer<br>Paternal Grandfather: Laryngeal Cancer<br>Paternal Grandmother: Stomach Cancer	*BRCA1*: c.188T>A; p.Leu63Ter<br>*BRCA2*: c.5576_5579del; p.Ile1859fs	19
BRCA1/*BRCA2*	Caucasian	UK	NI	Breast Cancer	FH not informative	*BRCA1*: c.211A>G; p.Arg71Gly<br>*BRCA2*: c.4380_4381del; p.Ser1461fs	20
BRCA1/*BRCA2*	Caucasian	Spain	NI	Breast Cancer	FH not informative	*BRCA1*: c.212+1G>A<br>*BRCA2*: c.739_740del; p.Ile247fs	20
BRCA1/*BRCA2*	Caucasian	Italy	NI	Breast Cancer	FH not informative	*BRCA1*: c.213-12A>G<br>*BRCA2*: c.7180A>T; p.Arg2394Ter	20
BRCA1/*BRCA2*	Scottish	Canada	35	Breast Cancer	Mother: Breast Cancer<br>Maternal Aunt: Breast Cancer<br>Maternal Uncle: Cancer of Unknown Origin<br>Maternal Grandmother: Breast Cancer<br>Maternal Grandfather: Colon Cancer<br>Paternal Grandmother: Breast Cancer<br>Paternal Nieces: Breast Cancer<br>Paternal Nieces: Breast Cancer<br>Paternal Nephew: Hodgkin's Lymphoma	*BRCA1*: c.2389G>T; p.Glu797Ter<br>*BRCA2*: c.3068dup; p.Asn1023fs	36
BRCA1/*BRCA2*	Italian	Italy	52<br>52	Breast Cancer<br>Ovarian Cancer	Sister: Breast and Ovarian Cancer<br>Paternal Grandmother: Cervix Cancer	*BRCA1*: c.2405_2406del; p.Val802fs	35
BRCA1/*BRCA2*	Caucasian	UK	NI	Breast Cancer	FH not informative	*BRCA1*: c.246del; p.Val83fs<br>*BRCA2*: c.517-2A>G	20

BRCA1/*BRCA2*	NI	Dutch	40<br>45	Ovarian Cancer<br>Breast Cancer	Mother: Ovarian Cancer<br>Father: Brain Tumor<br>Maternal Aunt: Bilateral Breast Cancer<br>Maternal Aunt: Stomach Cancer<br>Maternal Cousin: Breast Cancer	*BRCA1*: c.2685_2686del; p.Pro897fs<br>*BRCA2*: c.3487del; p.Asp1163fs	9
BRCA1/*BRCA2*	NI	Dutch	28	Breast Cancer	FH not informative	*BRCA1*: c.2685_2686del; p.Pro897fs<br>*BRCA2*: c.4449del; p.Asp1484fs	9
BRCA1/*BRCA2*	Caucasian	USA	NI	Breast Cancer	FH not informative	*BRCA1*: c.301+1G>A<br>*BRCA2*: c.5682C>G; p.Tyr1894Ter	20
BRCA1/*BRCA2*	NI	Sweden	NI	Breast Cancer	FH not informative	*BRCA1*: c.3048_3052dup; p.Asn1018fs<br>*BRCA2*: c.2830A>T; p.Lys944Ter	20
BRCA1/*BRCA2*	Caucasian	Australia	NI	Breast Cancer	FH not informative	*BRCA1*: c.3155del; p.Asn1052fs<br>*BRCA2*: c.3160_3163del; p.Asp1054fs	20
BRCA1/*BRCA2*	Caucasian	Germany	NI	Breast Cancer	FH not informative	*BRCA1*: c.3196G>T; p.Glu1066Ter<br>*BRCA2*: c.658_659del; p.Val220fs	20
BRCA1/*BRCA2*	Caucasian	Italy	NI	Breast Cancer	FH not informative	*BRCA1*: c.3228_3229del; p.Gly1077fs<br>*BRCA2*: c.9253dup; p.Thr3085fs	20
BRCA1/*BRCA2*	Caucasian	UK	NI	Breast Cancer	FH not informative	*BRCA1*: c.3400G>T; p.Glu1134Ter<br>*BRCA2*: c.2808_2811del; p.Ala938Profs	20
BRCA1/*BRCA2*	Korean	Korean	26	Breast Cancer	Mother: Bilateral Breast Cancer<br>Maternal Aunt: Bilateral Breast Cancer	*BRCA1*: c.3627dup; p.Glu1210fs<br>*BRCA2*: c.6724_6725del; p.Asp2242fs	17
BRCA1/*BRCA2*	Caucasian	Germany	40<br>26	Breast Cancer<br>Cervix Cancer	Sister: Cervix Cancer<br>Paternal Grandmother: Breast Cancer<br>Maternal Uncle: Skin and Prostate Cancer<br>Maternal Grandmother: Breast Cancer	*BRCA1*: c.3700_3704del; p.Val1234fs<br>*BRCA2*: c.1813dup; p.Ile605fs	27
BRCA1/*BRCA2*	Caucasian	Germany	NI	Breast Cancer	FH not informative	*BRCA1*: c.3700_3704del; p.Val1234fs<br>*BRCA2*: c.1815dupA; p.Pro606fs	20

BRCA1/*BRCA2*	Caucasian	Australia	NI	Breast Cancer	FH not informative	*BRCA1*: c.3700_3704del; p.Val1234fs<br>*BRCA2*: c.681+1G>A	20
BRCA1/*BRCA2*	Caucasian	USA	NI	Breast Cancer	FH not informative	*BRCA1*: c.3756_3759del; p.Val1234fs<br>*BRCA2*: c.7757G>A; p.Trp2586Ter	20
BRCA1/*BRCA2*	Australian	Australia	<40	Breast Cancer	FH not informative	*BRCA1*: c.3770_3771del; p.Glu1257fs<br>*BRCA2*: c.5946del; p.Ser1982fs	37
BRCA1/*BRCA2*	Korean	Korean	45	Breast Cancer	No Cancer	*BRCA1*: c.390C>A; p.Tyr130Ter<br>*BRCA2*: c.3018del; p.Gly1007fs	17
BRCA1/*BRCA2*	Caucasian	Germany	39	Breast Cancer	Mother: Breast Cancer<br>Maternal Aunt: Ovarian Cancer<br>Maternal Cousin: Breast Cancer	*BRCA1*: c.3910del; p.Glu1304fs<br>*BRCA2*: c.2830A>T; p.Lys944Ter	27
BRCA1/*BRCA2*	Italian	Italy	30<br>36	Breast Cancer<br>Ovarian Cancer	Father: Gastric Cancer<br>Mother: Cervix Cancer<br>(Mutation Negative)<br>Paternal Uncle: Lung Cancer<br>Paternal Aunt: Bilateral Breast Cancer<br>Paternal Aunt: Pancreatic Cancer	*BRCA1*: c.3916_3917del; p.Leu1306fs<br>*BRCA2*: c.5380del; p.Asn1793_Val1794insTer	35
BRCA1/*BRCA2*	Caucasian	USA	NI	Breast Cancer	FH not informative	*BRCA1*: c.4065_4068del; p.Asn1355fs<br>*BRCA2*: c.5350_5351del; p.Asn1784fs	20
BRCA1/*BRCA2*	Caucasian	UK	NI	Breast Cancer	FH not informative	*BRCA1*: c.4186-?_4357+?dup<br>*BRCA2*: c.2636_2637del; p.Asp878_Ser879insTer	20
BRCA1/*BRCA2*	Caucasian	Demark	NI	Breast Cancer	FH not informative	*BRCA1*: c.427G>T; p.Glu143Ter<br>*BRCA2*: c.8730delT; p.Asn2910fs	20
BRCA1/*BRCA2*	French Canadian	NI	52<br>63	Breast Cancer<br>Ovarian Cancer	FH not informative	*BRCA1*: c.4327C>T; p.Arg1443Ter<br>*BRCA2*: c.2588dup; p.Asn863fs	38
BRCA1/*BRCA2*	Korean	Korea	33	Breast Cancer	Mother: Stomach Cancer<br>Maternal Aunt: Larynx Cancer	*BRCA1*: c.4981G>T; p.Glu1661Ter<br>*BRCA2*: c.5946_5949del; p.Ser1982fs	18

BRCA1/*BRCA2*	Korean	Korea	35	Breast Cancer	Maternal Grandmother: Ovary Cancer<br>Maternal Aunt: Esophageal Cancer	*BRCA1*: c.5030_5033del; p.Thr1677fs<br>*BRCA2*: c.1399A>T; p.Lys467Ter	17
BRCA1/*BRCA2*	Caucasian	Germany	NI	Breast Cancer	FH not informative	*BRCA1*: c.5123C>A; p.Ala1708Glu<br>*BRCA2*: c.6275_6276del; c.6275_6276del p.Leu2092fs	20
BRCA1/*BRCA2*	Asian	USA	NI	Breast Cancer	FH not informative	*BRCA1*: c.5136G>A; p.Leu2092fs<br>*BRCA2*: c.4965del; p.Cys1654_Tyr1655insTer	20
BRCA1/*BRCA2*	Caucasian	Austria	NI	Breast Cancer	FH not informative	*BRCA1*: c.5251C>T; p.Arg1751Ter<br>*BRCA2*: c.6753_6754del; p.Leu2253fs	20
BRCA1/*BRCA2*	Caucasian	Germany	37<br>63	Bilateral Breast Cancer<br>Bilateral Ovarian Cancer	Father: Prostate Cancer<br>Paternal Grandmother: Breast Cancer<br>Paternal Grandaunt: Breast Cancer<br>Nieces: Breast Cancer	*BRCA1*: c.5266dup; p.Gln1756fs<br>*BRCA2*: c.5645C>A; p.Ser1882Ter	27
BRCA1/*BRCA2*	Caucasian	Italy	38<br>42	Breast Cancer<br>Ovarian Cancer	Breast Cancer<br>Ovarian Cancer	*BRCA1*: c.5266dup; p.Gln1756fs<br>*BRCA2*: c.5796_5797del; p.His1932fs	39
BRCA1/*BRCA2*	NI	Netherlands	51	Breast Cancer	Brother's Daughter: Breast Cancer<br>Maternal Aunt: Breast Cancer<br>Maternal Cousin: Colon Cancer<br>Paternal Cousin: Cancer of Unknown Origin	*BRCA1*: c.5266dup; p.Gln1756fs<br>*BRCA2*: c.5946del; p.Ser1982fs	9
BRCA1/*BRCA2*	Caucasian	Austria	NI	Breast Cancer	FH not informative	*BRCA1*: c.5266dup; p.Gln1756fs<br>*BRCA2*: c.8364G>A; p.Trp2788Ter	20
BRCA1/*BRCA2*	Caucasian	Germany	NI	Breast Cancer	FH not informative	*BRCA1*: c.5266dupC; p.Gln1756fs<br>*BRCA2*: c.4478_4481del; p.Glu1493fs	20
BRCA1/*BRCA2*	Caucasian	Germany	NI<br>NI	Breast Cancer<br>Ovarian Cancer	FH not informative	*BRCA1*: c.5266dupC; p.Gln1756fs<br>*BRCA2*: c.5645C>A; p.Ser1882Ter	20
BRCA1/*BRCA2*	Caucasian	Greece	NI	Breast Cancer	FH not informative	*BRCA1*: c.5406+664_*8273del<br>*BRCA2*: c.9748dup; p.Ser3250fs	20

BRCA1/*BRCA2*	Caucasian	Germany	NI	Breast Cancer<br>Ovarian Cancer	FH not informative	*BRCA1*: c.548-?_4185+?del<br>*BRCA2*: c.2269A>T; p.Lys757Ter	20
BRCA1/*BRCA2*	Caucasian	Germany	32	Breast Cancer	Mother: Ovarian Cancer<br>Maternal Grandmother: Breast Cancer<br>Maternal Grandaunt: Breast Cancer<br>Father: Prostate Cancer<br>Paternal Aunt: Bilateral Breast Cancer<br>Paternal Aunt: Breast Cancer<br>Paternal Aunt: Breast Cancer<br>Paternal Uncle: Prostate & Testicular Cancer<br>Paternal Grandmother: Pancreas Cancer<br>Paternal Grandfather: Liver Cancer<br>Paternal Grandaunt: Breast Cancer	*BRCA1*: c.68_69del; p.Glu23fs<br>*BRCA2*: c.5722_5723del; p.Leu1908fs	27
BRCA1/*BRCA2*	NI	USA<br>Hungary<br>Israel	NI	13 Breast Cancers<br>3 Breast & Ovarian Cancers	FH not informative	*BRCA1*: c.68_69del; p.Glu23fs<br>*BRCA2*: c.5946del; p.Ser1982fs	20
BRCA1/*BRCA2*	Italian	Italy	43	Breast Cancer	Mother: Bilateral Breast Cancer<br>Maternal Uncle: Leukemia<br>Maternal Grand Mother: Breast Cancer	*BRCA1*: c.834_835insA; p.His279fs<br>*BRCA2*: c.8195T>A; p.Leu2732Ter	35
BRCA1/*BRCA2*	Caucasian	Germany	31	Bilateral Breast Cancer	Sister: Breast Cancer<br>Mother: Breast Cancer<br>Maternal Grandmother: Breast Cancer<br>Maternal Aunt: Breast Cancer	*BRCA1*: c.962G>A; p.Trp321Ter<br>*BRCA2*: c.2231C>G; p.Ser744Ter	27
BRCA1/*BRCA2*	Caucasian	Germany	NI	Breast Cancer	FH not informative	*BRCA1*: c.962G>A; p.Trp321Ter<br>*BRCA2*: c.2231C>G; p.Ser744Ter	20

BRCA1/MLH1	Caucasian	Italy	39<br><br>39<br><br>39<br>35<br>46	Endometrial Cancer<br>Ovarian Cancer<br>Kidney Cancer<br>Breast Cancer<br>Breast Cancer	Son: Brain Cancer<br>Mother: Colon Cancer, Endometrial Cancer, Thyroid Cancer<br>Maternal Aunt: Colon Cancer<br>Maternal Aunt: Colon Cancer<br>Maternal Uncle: Colon Cancer<br>Maternal Cousin: Ovarian Cancer<br>Maternal Cousin: Gastric Cancer<br>Paternal Uncle: Pancreatic Cancer<br>Paternal Cousin: Colon Cancer<br>Paternal Grandmother: Breast Cancer	*BRCA1*: c.181T>G; p.Cys61Gly<br>*MLH1*: c.1489dup; p.Arg497Profs*6	40
BRCA1/TP53	NI	Canada	20	Breast Cancer	Mother: Cervical Cancer<br>Maternal Grandmother: Lung and Brain Cancer<br>Maternal Aunt: Breast Cancer	*BRCA1*: c.81-?_134 ? Del; p.Cys27*<br>*TP53*: c.375+2T>C	41
BRCA2/STK11	Iranian	Iran	41	Breast Cancer	Sister: Breast Cancer<br>Sister: Breast Cancer<br>Paternal Aunt: Breast Cancer	*BRCA2*: c.2808_2811del; p.936_937del<br>*STK11*: c.1264A>G; p.Ser422Gly	42
BRCA2/MSH2	NI	Canada	32	Breast Cancer	Sister: Breast Cancer<br>Father: Colon Cancer<br>Paternal Uncle: Colon Cancer<br>Paternal Uncle: Multiple Myeloma<br>Paternal Uncle: Lung Cancer<br>Paternal Uncle: Lung Cancer<br>Paternal Uncle: Rectum and Brain Cancers<br>Paternal Aunt: Ovarian Cancer<br>Paternal Aunt: Uterus Cancer	*BRCA2*: c.314T>G; p.Leu105Ter<br>*MSH2*: exon 8 deletion	43

BRCA2/PALB2	French Canadian	Canada	45	Breast Cancer	Father: Lung Cancer<br>Paternal Aunt: Breast Cancer<br>Paternal Aunt: Neuroendocrine Tumor<br>Paternal Aunt: Esophageal Cancer<br>Paternal Uncle: Lung Cancer<br>Paternal Uncle: Lung and Rectum Cancer<br>Paternal Uncle: Bladder Cancer<br>Maternal Aunt: Breast Cancer<br>Maternal Aunt: Breast Cancer<br>Maternal Aunt: Breast Cancer<br>Maternal Aunt: Colon Cancer<br>Maternal Aunt: Colon Cancer<br>Maternal Aunt: Colon Cancer	*PALB2*: c.2323C>T; p.Gln775Ter<br>*BRCA2*: c.9004G>A; p.Glu3002Lys	44
ATM/CHEK2	Slavic	Belarus	67	Breast Cancer	No Cancer	*ATM*: c.5932G>T; p.Glu1978Ter<br>*CHEK2*: Del exon 9-10; NC_000022.10:g.27416941_27422508del	12
BLM/CHEK2	Slavic	Russia	51	Breast Cancer	Mother: Leukemia	*CHEK2*: c.1100del; p.Thr367fs<br>*BLM*: c.1642C>T; p.Gln548Ter	12
BLM/CHEK2	Slavic	Belarus	44	Breast Cancer	No Cancer	*CHEK2*: Del exon 9-10; NC_000022.10:g.27416941_27422508del<br>*BLM*: c.1642C>T; p.Gln548Ter	12
BLM/NBS1	Slavic	Russia	48	Breast Cancer	Grandmother: Colon Cancer	*BLM*: c.1642C>T; p.Gln548Ter<br>*NBN*: c.657_661del (p.Lys219fs	12
CHEK2/NBN	Slavic	Belarus	53	Breast Cancer	No Cancer	*CHEK2*: c.444+1G>A<br>*NBN*: c.657_661del; p.Lys219fs	12
CHEK2/NBN	Slavic	Poland	59	Bilateral Breast Cancer	No Cancer	*CHEK2*: c.444+1G>A<br>*NBN*: c.657_661del; p.Lys219fs	12
CHEK2/NBN	Slavic	Poland	53	Breast Cancer	No Cancer	*CHEK2*: c.444+1G>A<br>*NBN*: c.657_661del; p.Lys219fs	12

NI: no information provided
